# Supplementary material for: From sequence to enzyme mechanism using multi-label machine learning
Source: BMC Bioinformatics. 2014 May 19;15:150. doi: 10.1186/1471-2105-15-150 (PMC4229970; doi:10.1186/1471-2105-15-150)
Supplement: Additional file 2 — Java code of ml2db. Additional file ml2db_code.tar.gz contains the Java source code to run the multi-label machine learning experiments and save the results to database. The code’s Javadoc is included. [file 1471-2105-15-150-S2.zip › additional file 2/ml2db/utils/doc/uk/ac/standrews/utils/main/maputils/MapOfMaps.html]

MapOfMaps


JavaScript is disabled on your browser.


- Overview
- Package
- Class
- Use
- Tree
- Deprecated
- Index
- Help

- Prev Class
- Next Class

- Frames
- No Frames

- All Classes

- Summary:
- Nested |
- Field |
- Constr |
- Method

- Detail:
- Field |
- Constr |
- Method


uk.ac.standrews.utils.main.maputils

## Class MapOfMaps

- java.lang.Object
- - java.util.AbstractMap<K,V>
  - - java.util.TreeMap<java.lang.String,java.util.Map<java.lang.String,java.lang.String>>
    - - uk.ac.standrews.utils.main.maputils.MapOfMaps

- All Implemented Interfaces:
  :   java.io.Serializable, java.lang.Cloneable, java.util.Map<java.lang.String,java.util.Map<java.lang.String,java.lang.String>>, java.util.NavigableMap<java.lang.String,java.util.Map<java.lang.String,java.lang.String>>, java.util.SortedMap<java.lang.String,java.util.Map<java.lang.String,java.lang.String>>

  ---

    

  ```
  public class MapOfMaps
  extends java.util.TreeMap<java.lang.String,java.util.Map<java.lang.String,java.lang.String>>
  ```

  A map of maps. Each key (referred to as external key) has as value a map,
  that is a collection of (internal) key-value tuples.
  Uses string pools to save memory. Do not modify any string after import. This
  is supposed to be an add-only, read-only kind of object for moving data from
  database to other structures.

  Author:
  :   Luna De Ferrari ldf@st-andrews.ac.uk

  See Also:
  :   Serialized Form

- - ### Nested Class Summary

    - ### Nested classes/interfaces inherited from class java.util.AbstractMap

      `java.util.AbstractMap.SimpleEntry<K,V>, java.util.AbstractMap.SimpleImmutableEntry<K,V>`
  - ### Constructor Summary

    Constructors

    Constructor and Description

    `MapOfMaps(boolean acceptEmptyStrings)`

    `MapOfMaps(boolean acceptEmptyStrings,
    int uniqueExtKeys,
    int uniqueIntKeys,
    int uniqueValues)`

    `MapOfMaps(boolean acceptEmptyStrings,
    java.util.Vector<java.lang.String> extKeys,
    java.util.Vector<java.lang.String> intKeys,
    java.util.Vector<java.lang.String> values)`

    `MapOfMaps(boolean acceptEmptyStrings,
    java.util.Vector<java.lang.String> extKeys,
    java.util.Vector<java.lang.String> intKeys,
    java.util.Vector<java.lang.String> values,
    int uniqueExtKeys,
    int uniqueIntKeys,
    int uniqueValues)`
  - ### Method Summary

    Methods

    | Modifier and Type | Method and Description |
    | --- | --- |
    | `boolean` | `acceptEmptyStrings()` |
    | `void` | `addAll(MapOfMaps anotherMap)` |
    | `boolean` | `addKeyKeyValue(java.lang.String extkey, java.lang.String intkey, java.lang.String value)` Add a key-key-value tuple to the map |
    | `boolean` | `containsInternalKeyValue(java.lang.String aKey, java.lang.String aValue)` |
    | `boolean` | `containsKeyKeyValue(java.lang.String extkey, java.lang.String intkey, java.lang.String value)` Checks if the map contains a specific [external key, internal key, value] triple |
    | `int` | `countInternalKeys()` Get how many unique internal keys are there in all the internal maps |
    | `void` | `generateInternalKeys()` |
    | `OneToManyMap<java.lang.String,java.lang.String>` | `getExternalInternalKeyMap()` A one to many map containing for each external key the list of unique internal keys in its map. |
    | `StringPool` | `getExtStringPool()` |
    | `java.util.TreeSet<java.lang.String>` | `getInternalKeys()` |
    | `StringPool` | `getIntStringPool()` |
    | `java.util.TreeSet<java.lang.String>` | `getUniqueValues()` Get the set of unique values from all internal maps |
    | `StringPool` | `getValuesStringPool()` |
    | `java.lang.String` | `toString()` |

    - ### Methods inherited from class java.util.TreeMap

      `ceilingEntry, ceilingKey, clear, clone, comparator, containsKey, containsValue, descendingKeySet, descendingMap, entrySet, firstEntry, firstKey, floorEntry, floorKey, get, headMap, headMap, higherEntry, higherKey, keySet, lastEntry, lastKey, lowerEntry, lowerKey, navigableKeySet, pollFirstEntry, pollLastEntry, put, putAll, remove, size, subMap, subMap, tailMap, tailMap, values`
    - ### Methods inherited from class java.util.AbstractMap

      `equals, hashCode, isEmpty`
    - ### Methods inherited from class java.lang.Object

      `getClass, notify, notifyAll, wait, wait, wait`
    - ### Methods inherited from interface java.util.Map

      `equals, hashCode, isEmpty`

- - ### Constructor Detail


    - #### MapOfMaps

      ```
      public MapOfMaps(boolean acceptEmptyStrings)
      ```


    - #### MapOfMaps

      ```
      public MapOfMaps(boolean acceptEmptyStrings,
               int uniqueExtKeys,
               int uniqueIntKeys,
               int uniqueValues)
      ```


    - #### MapOfMaps

      ```
      public MapOfMaps(boolean acceptEmptyStrings,
               java.util.Vector<java.lang.String> extKeys,
               java.util.Vector<java.lang.String> intKeys,
               java.util.Vector<java.lang.String> values)
      ```


    - #### MapOfMaps

      ```
      public MapOfMaps(boolean acceptEmptyStrings,
               java.util.Vector<java.lang.String> extKeys,
               java.util.Vector<java.lang.String> intKeys,
               java.util.Vector<java.lang.String> values,
               int uniqueExtKeys,
               int uniqueIntKeys,
               int uniqueValues)
      ```
  - ### Method Detail


    - #### acceptEmptyStrings

      ```
      public boolean acceptEmptyStrings()
      ```


    - #### addAll

      ```
      public void addAll(MapOfMaps anotherMap)
      ```


    - #### addKeyKeyValue

      ```
      public boolean addKeyKeyValue(java.lang.String extkey,
                           java.lang.String intkey,
                           java.lang.String value)
      ```

      Add a key-key-value tuple to the map

      Parameters:
      :   `extkey` - external key
      :   `intkey` - internal key
      :   `value` - a value

      Returns:
      :   true if the value was added, false if the key-key-value pair
          already existed


    - #### generateInternalKeys

      ```
      public void generateInternalKeys()
      ```


    - #### containsInternalKeyValue

      ```
      public boolean containsInternalKeyValue(java.lang.String aKey,
                                     java.lang.String aValue)
      ```


    - #### containsKeyKeyValue

      ```
      public boolean containsKeyKeyValue(java.lang.String extkey,
                                java.lang.String intkey,
                                java.lang.String value)
      ```

      Checks if the map contains a specific [external key, internal key, value]
      triple

      Parameters:
      :   `extkey` -
      :   `intkey` -
      :   `value` -

      Returns:
      :   true if the key-key-value exists, false otherwise (false if the
          keys or value are null)


    - #### countInternalKeys

      ```
      public int countInternalKeys()
      ```

      Get how many unique internal keys are there in all the internal maps


    - #### getExternalInternalKeyMap

      ```
      public OneToManyMap<java.lang.String,java.lang.String> getExternalInternalKeyMap()
      ```

      A one to many map containing for each external key the list of unique
      internal keys in its map.

      Returns:


    - #### getExtStringPool

      ```
      public StringPool getExtStringPool()
      ```


    - #### getInternalKeys

      ```
      public java.util.TreeSet<java.lang.String> getInternalKeys()
      ```


    - #### getIntStringPool

      ```
      public StringPool getIntStringPool()
      ```


    - #### getUniqueValues

      ```
      public java.util.TreeSet<java.lang.String> getUniqueValues()
      ```

      Get the set of unique values from all internal maps

      Returns:


    - #### getValuesStringPool

      ```
      public StringPool getValuesStringPool()
      ```


    - #### toString

      ```
      public java.lang.String toString()
      ```

      **Overrides:**
      :   `toString` in class `java.util.AbstractMap<java.lang.String,java.util.Map<java.lang.String,java.lang.String>>`


- Overview
- Package
- Class
- Use
- Tree
- Deprecated
- Index
- Help

- Prev Class
- Next Class

- Frames
- No Frames

- All Classes

- Summary:
- Nested |
- Field |
- Constr |
- Method

- Detail:
- Field |
- Constr |
- Method
